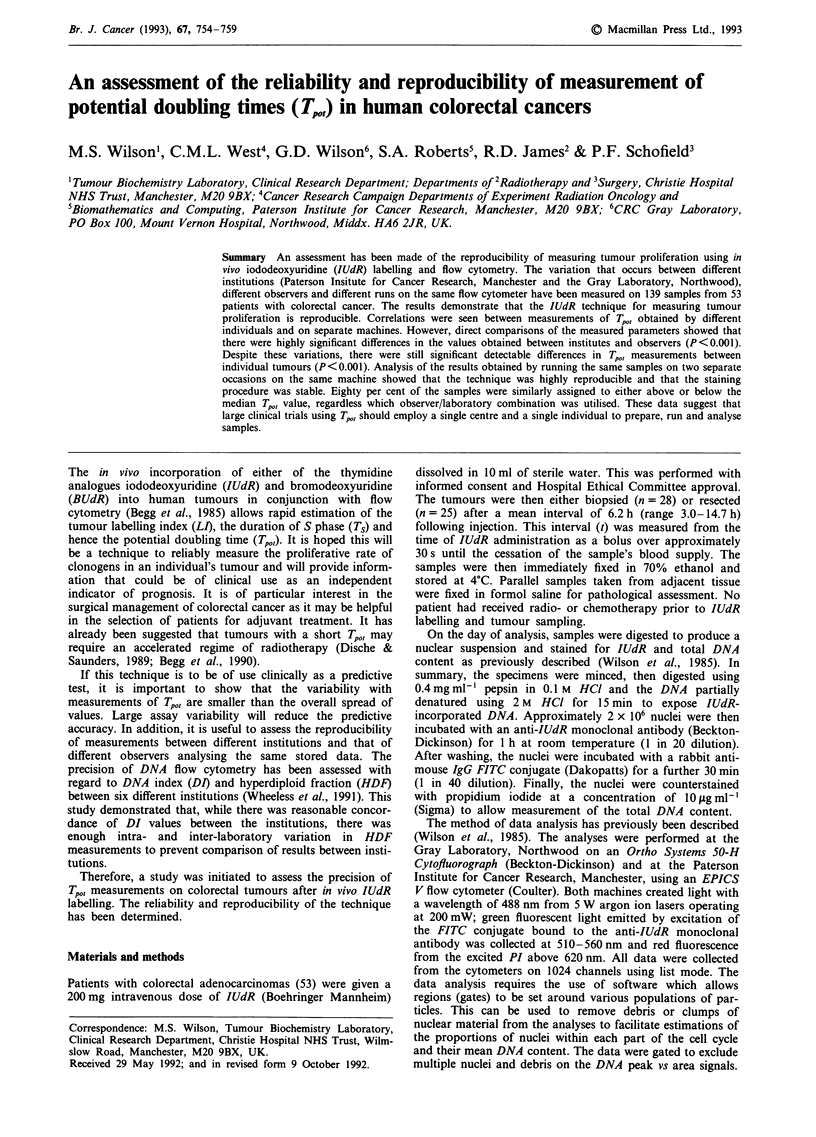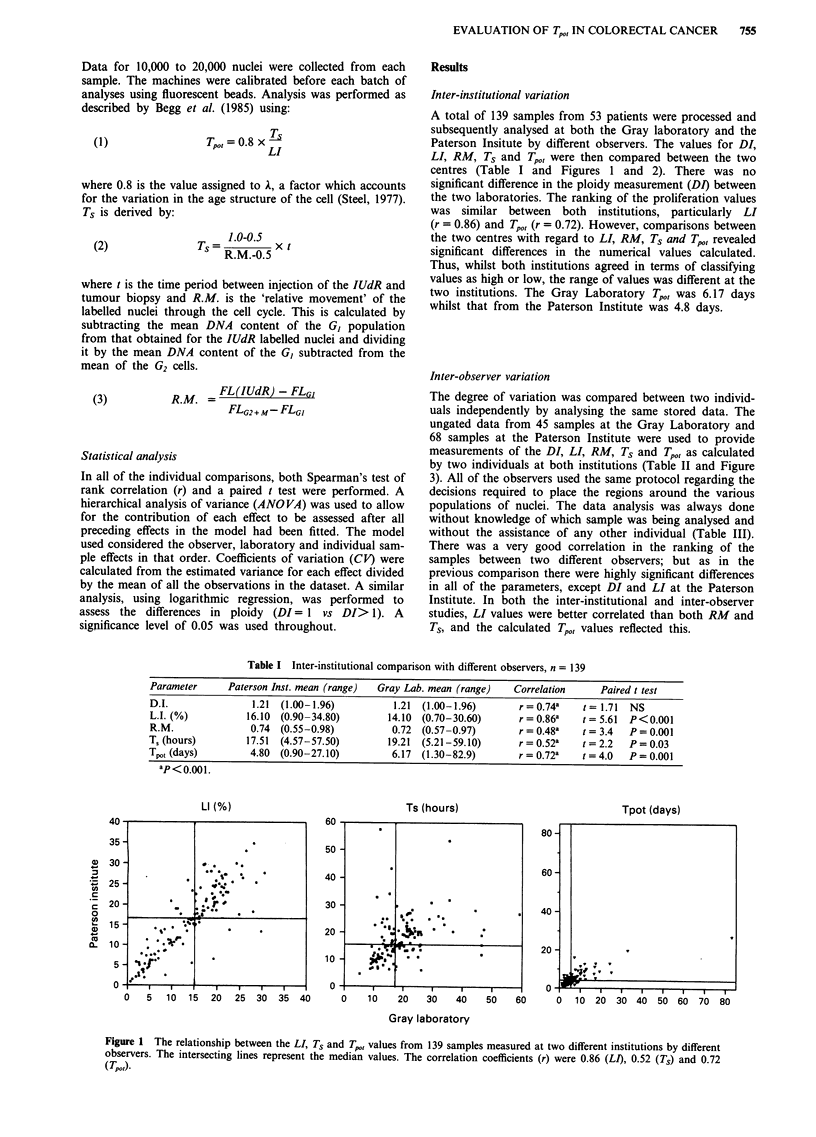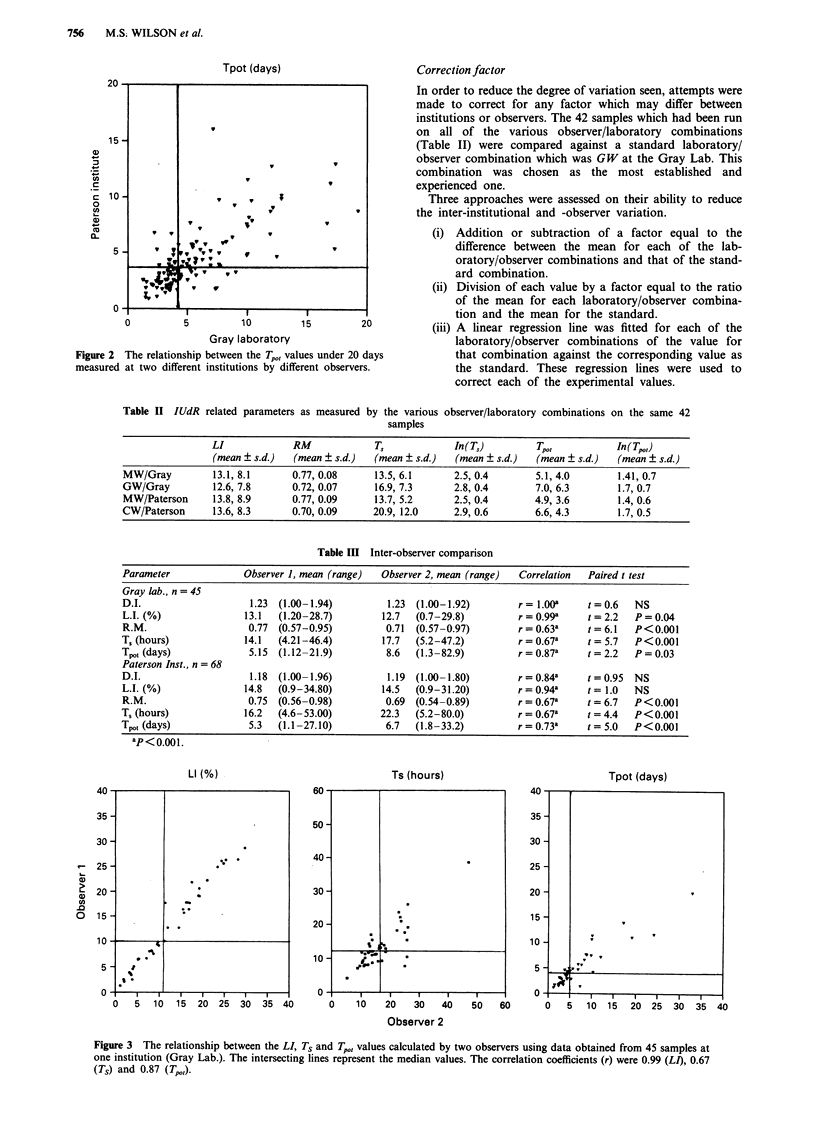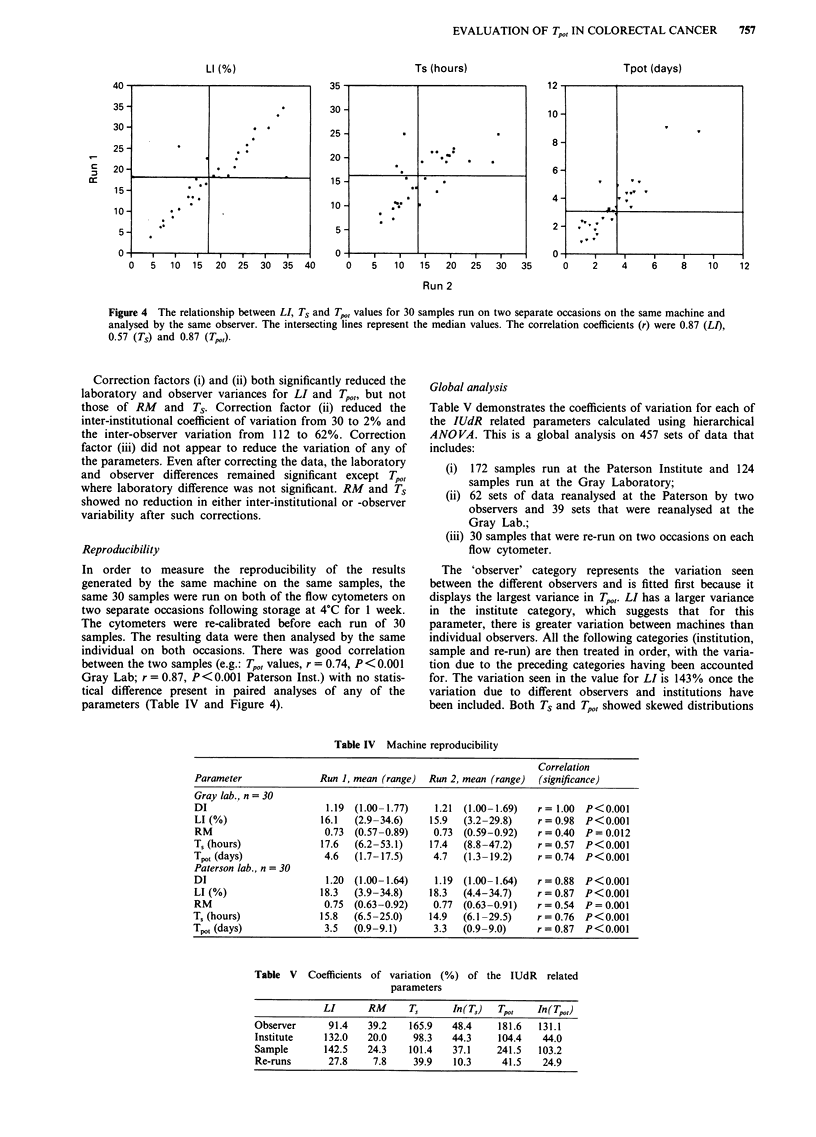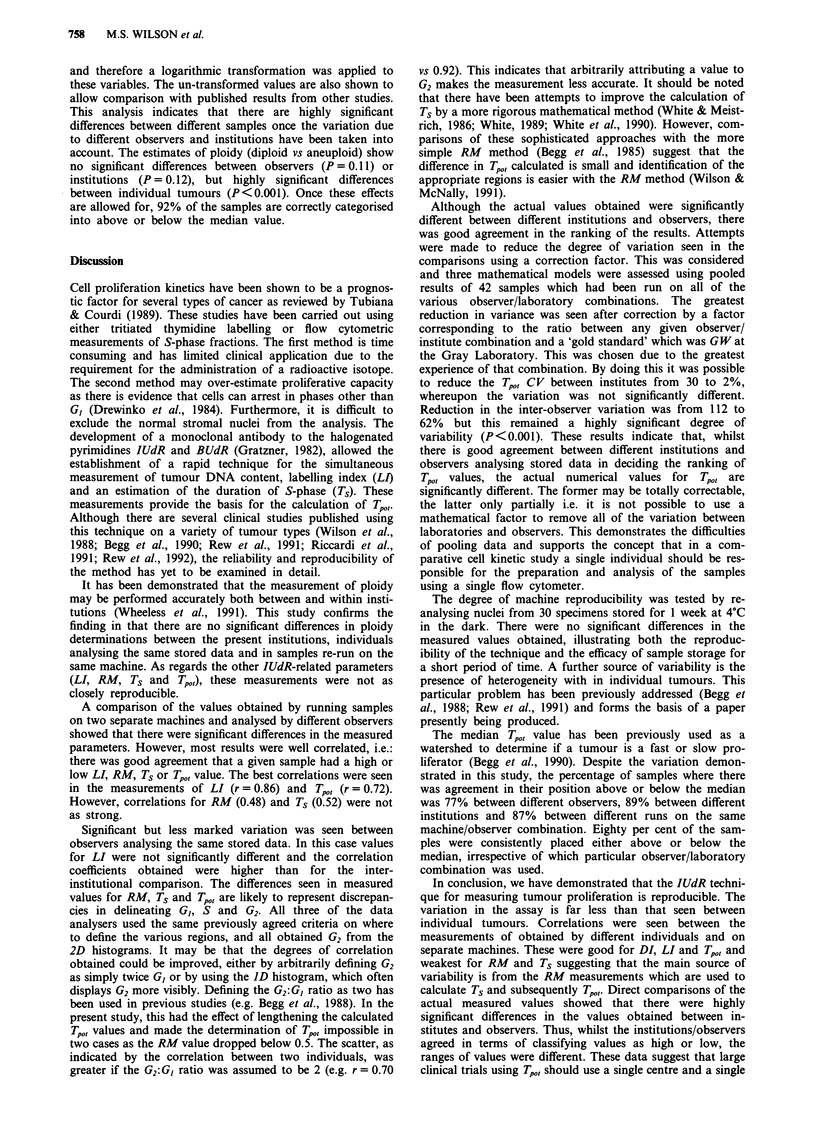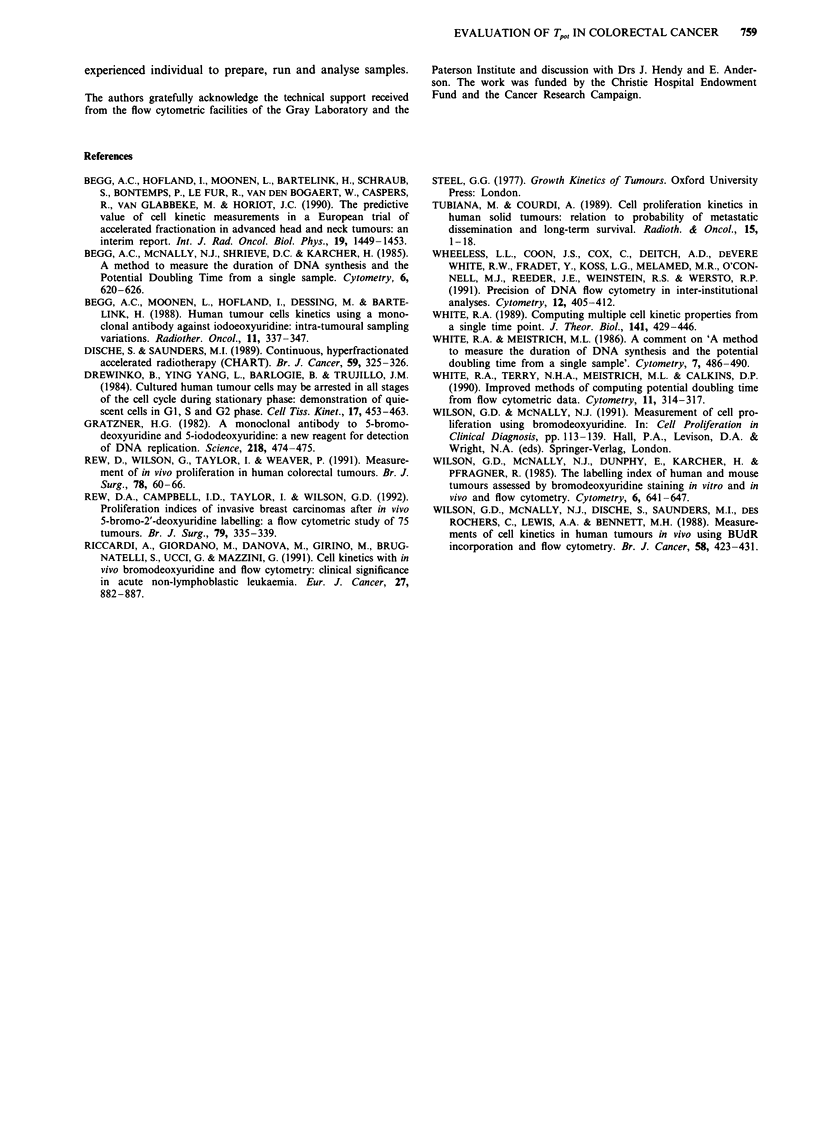# An assessment of the reliability and reproducibility of measurement of potential doubling times (Tpot) in human colorectal cancers.

**DOI:** 10.1038/bjc.1993.137

**Published:** 1993-04

**Authors:** M. S. Wilson, C. M. West, G. D. Wilson, S. A. Roberts, R. D. James, P. F. Schofield

**Affiliations:** Clinical Research Department, Christie Hospital, Manchester, UK.

## Abstract

An assessment has been made of the reproducibility of measuring tumour proliferation using in vivo iododeoxyuridine (IUdR) labelling and flow cytometry. The variation that occurs between different institutions (Paterson Institute for Cancer Research, Manchester and the Gray Laboratory, Northwood), different observers and different runs on the same flow cytometer have been measured on 139 samples from 53 patients with colorectal cancer. The results demonstrate that the IUdR technique for measuring tumour proliferation is reproducible. Correlations were seen between measurements of Tpot obtained by different individuals and on separate machines. However, direct comparisons of the measured parameters showed that there were highly significant differences in the values obtained between institutes and observers (P < 0.001). Despite these variations, there were still significant detectable differences in Tpot measurements between individual tumours (P < 0.001). Analysis of the results obtained by running the same samples on two separate occasions on the same machine showed that the technique was highly reproducible and that the staining procedure was stable. Eighty per cent of the samples were similarly assigned to either above or below the median Tpot value, regardless which observer/laboratory combination was utilised. These data suggest that large clinical trials using Tpot should employ a single centre and a single individual to prepare, run and analyse samples.


Br. J. Cancer (1993), 67, 754–759 © Macmillan Press Ltd., 1993

### An assessment of the reliability and reproducibility of measurement of potential doubling times ($T_{pot}$) in human colorectal cancers

M.S. Wilson[1], C.M.L. West[4], G.D. Wilson[6], S.A. Roberts[5], R.D. James[2] & P.F. Schofield[3]

[1]*Tumour Biochemistry Laboratory, Clinical Research Department; Departments of* [2]*Radiotherapy and* [3]*Surgery, Christie Hospital NHS Trust, Manchester, M20 9BX;* [4]*Cancer Research Campaign Departments of Experiment Radiation Oncology and* [5]*Biomathematics and Computing, Paterson Institute for Cancer Research, Manchester, M20 9BX;* [6]*CRC Gray Laboratory, PO Box 100, Mount Vernon Hospital, Northwood, Middx. HA6 2JR, UK.*

**Summary** An assessment has been made of the reproducibility of measuring tumour proliferation using *in vivo* iododeoxyuridine (*IUdR*) labelling and flow cytometry. The variation that occurs between different institutions (Paterson Insitute for Cancer Research, Manchester and the Gray Laboratory, Northwood), different observers and different runs on the same flow cytometer have been measured on 139 samples from 53 patients with colorectal cancer. The results demonstrate that the *IUdR* technique for measuring tumour proliferation is reproducible. Correlations were seen between measurements of $T_{pot}$ obtained by different individuals and on separate machines. However, direct comparisons of the measured parameters showed that there were highly significant differences in the values obtained between institutes and observers ($P < 0.001$). Despite these variations, there were still significant detectable differences in $T_{pot}$ measurements between individual tumours ($P < 0.001$). Analysis of the results obtained by running the same samples on two separate occasions on the same machine showed that the technique was highly reproducible and that the staining procedure was stable. Eighty per cent of the samples were similarly assigned to either above or below the median $T_{pot}$ value, regardless which observer/laboratory combination was utilised. These data suggest that large clinical trials using $T_{pot}$ should employ a single centre and a single individual to prepare, run and analyse samples.

The *in vivo* incorporation of either of the thymidine analogues iododeoxyuridine (*IUdR*) and bromodeoxyuridine (*BUdR*) into human tumours in conjunction with flow cytometry (Begg *et al.*, 1985) allows rapid estimation of the tumour labelling index (*LI*), the duration of *S* phase ($T_S$) and hence the potential doubling time ($T_{pot}$). It is hoped this will be a technique to reliably measure the proliferative rate of clonogens in an individual's tumour and will provide information that could be of clinical use as an independent indicator of prognosis. It is of particular interest in the surgical management of colorectal cancer as it may be helpful in the selection of patients for adjuvant treatment. It has already been suggested that tumours with a short $T_{pot}$ may require an accelerated regime of radiotherapy (Dische & Saunders, 1989; Begg *et al.*, 1990).

If this technique is to be of use clinically as a predictive test, it is important to show that the variability with measurements of $T_{pot}$ are smaller than the overall spread of values. Large assay variability will reduce the predictive accuracy. In addition, it is useful to assess the reproducibility of measurements between different institutions and that of different observers analysing the same stored data. The precision of *DNA* flow cytometry has been assessed with regard to *DNA* index (*DI*) and hyperdiploid fraction (*HDF*) between six different institutions (Wheeless *et al.*, 1991). This study demonstrated that, while there was reasonable concordance of *DI* values between the institutions, there was enough intra- and inter-laboratory variation in *HDF* measurements to prevent comparison of results between institutions.

Therefore, a study was initiated to assess the precision of $T_{pot}$ measurements on colorectal tumours after *in vivo IUdR* labelling. The reliability and reproducibility of the technique has been determined.

### Materials and methods

Patients with colorectal adenocarcinomas (53) were given a 200 mg intravenous dose of *IUdR* (Boehringer Mannheim) dissolved in 10 ml of sterile water. This was performed with informed consent and Hospital Ethical Committee approval. The tumours were then either biopsied ($n = 28$) or resected ($n = 25$) after a mean interval of 6.2 h (range 3.0–14.7 h) following injection. This interval ($t$) was measured from the time of *IUdR* administration as a bolus over approximately 30 s until the cessation of the sample's blood supply. The samples were then immediately fixed in 70% ethanol and stored at 4°C. Parallel samples taken from adjacent tissue were fixed in formol saline for pathological assessment. No patient had received radio- or chemotherapy prior to *IUdR* labelling and tumour sampling.

On the day of analysis, samples were digested to produce a nuclear suspension and stained for *IUdR* and total *DNA* content as previously described (Wilson *et al.*, 1985). In summary, the specimens were minced, then digested using 0.4 mg ml$^{-1}$ pepsin in 0.1 M *HCl* and the *DNA* partially denatured using 2 M *HCl* for 15 min to expose *IUdR*-incorporated *DNA*. Approximately $2 \times 10^6$ nuclei were then incubated with an anti-*IUdR* monoclonal antibody (Beckton-Dickinson) for 1 h at room temperature (1 in 20 dilution). After washing, the nuclei were incubated with a rabbit anti-mouse *IgG FITC* conjugate (Dakopatts) for a further 30 min (1 in 40 dilution). Finally, the nuclei were counterstained with propidium iodide at a concentration of 10 $\mu$g ml$^{-1}$ (Sigma) to allow measurement of the total *DNA* content.

The method of data analysis has previously been described (Wilson *et al.*, 1985). The analyses were performed at the Gray Laboratory, Northwood on an *Ortho Systems 50-H Cytofluorograph* (Beckton-Dickinson) and at the Paterson Institute for Cancer Research, Manchester, using an *EPICS V* flow cytometer (Coulter). Both machines created light with a wavelength of 488 nm from 5 W argon ion lasers operating at 200 mW; green fluorescent light emitted by excitation of the *FITC* conjugate bound to the anti-*IUdR* monoclonal antibody was collected at 510–560 nm and red fluorescence from the excited *PI* above 620 nm. All data were collected from the cytometers on 1024 channels using list mode. The data analysis requires the use of software which allows regions (gates) to be set around various populations of particles. This can be used to remove debris or clumps of nuclear material from the analyses to facilitate estimations of the proportions of nuclei within each part of the cell cycle and their mean *DNA* content. The data were gated to exclude multiple nuclei and debris on the *DNA* peak *vs* area signals.

Correspondence: M.S. Wilson, Tumour Biochemistry Laboratory, Clinical Research Department, Christie Hospital NHS Trust, Wilmslow Road, Manchester, M20 9BX, UK.
Received 29 May 1992; and in revised form 9 October 1992.

Data for 10,000 to 20,000 nuclei were collected from each sample. The machines were calibrated before each batch of analyses using fluorescent beads. Analysis was performed as described by Begg *et al.* (1985) using:

$$(1) \qquad T_{pot} = 0.8 \times \frac{T_S}{LI}$$

where 0.8 is the value assigned to λ, a factor which accounts for the variation in the age structure of the cell (Steel, 1977). $T_S$ is derived by:

$$(2) \qquad T_S = \frac{1.0\text{-}0.5}{\text{R.M.-}0.5} \times t$$

where $t$ is the time period between injection of the *IUdR* and tumour biopsy and *R.M.* is the 'relative movement' of the labelled nuclei through the cell cycle. This is calculated by subtracting the mean *DNA* content of the $G_1$ population from that obtained for the *IUdR* labelled nuclei and dividing it by the mean *DNA* content of the $G_1$ subtracted from the mean of the $G_2$ cells.

$$(3) \qquad R.M. = \frac{FL(IUdR) - FL_{G1}}{FL_{G2+M} - FL_{G1}}$$

### *Statistical analysis*

In all of the individual comparisons, both Spearman's test of rank correlation ($r$) and a paired $t$ test were performed. A hierarchical analysis of variance (*ANOVA*) was used to allow for the contribution of each effect to be assessed after all preceding effects in the model had been fitted. The model used considered the observer, laboratory and individual sample effects in that order. Coefficients of variation (*CV*) were calculated from the estimated variance for each effect divided by the mean of all the observations in the dataset. A similar analysis, using logarithmic regression, was performed to assess the differences in ploidy ($DI = 1$ *vs* $DI > 1$). A significance level of 0.05 was used throughout.

### Results

### *Inter-institutional variation*

A total of 139 samples from 53 patients were processed and subsequently analysed at both the Gray laboratory and the Paterson Insitute by different observers. The values for *DI*, *LI*, *RM*, $T_S$ and $T_{pot}$ were then compared between the two centres (Table I and Figures 1 and 2). There was no significant difference in the ploidy measurement (*DI*) between the two laboratories. The ranking of the proliferation values was similar between both institutions, particularly *LI* ($r = 0.86$) and $T_{pot}$ ($r = 0.72$). However, comparisons between the two centres with regard to *LI*, *RM*, $T_S$ *and* $T_{pot}$ revealed significant differences in the numerical values calculated. Thus, whilst both institutions agreed in terms of classifying values as high or low, the range of values was different at the two institutions. The Gray Laboratory $T_{pot}$ was 6.17 days whilst that from the Paterson Institute was 4.8 days.

### *Inter-observer variation*

The degree of variation was compared between two individuals independently by analysing the same stored data. The ungated data from 45 samples at the Gray Laboratory and 68 samples at the Paterson Institute were used to provide measurements of the *DI*, *LI*, *RM*, $T_S$ and $T_{pot}$ as calculated by two individuals at both institutions (Table II and Figure 3). All of the observers used the same protocol regarding the decisions required to place the regions around the various populations of nuclei. The data analysis was always done without knowledge of which sample was being analysed and without the assistance of any other individual (Table III). There was a very good correlation in the ranking of the samples between two different observers; but as in the previous comparison there were highly significant differences in all of the parameters, except *DI* and *LI* at the Paterson Institute. In both the inter-institutional and inter-observer studies, *LI* values were better correlated than both *RM* and $T_S$, and the calculated $T_{pot}$ values reflected this.

**Table I** Inter-institutional comparison with different observers, $n = 139$

| *Parameter* | *Paterson Inst. mean (range)* | *Gray Lab. mean (range)* | *Correlation* | *Paired t test* |
|---|---|---|---|---|
| D.I. | 1.21 (1.00–1.96) | 1.21 (1.00–1.96) | $r = 0.74^a$ | $t = 1.71$ NS |
| L.I. (%) | 16.10 (0.90–34.80) | 14.10 (0.70–30.60) | $r = 0.86^a$ | $t = 5.61$ $P < 0.001$ |
| R.M. | 0.74 (0.55–0.98) | 0.72 (0.57–0.97) | $r = 0.48^a$ | $t = 3.4$ $P = 0.001$ |
| $T_s$ (hours) | 17.51 (4.57–57.50) | 19.21 (5.21–59.10) | $r = 0.52^a$ | $t = 2.2$ $P = 0.03$ |
| $T_{pot}$ (days) | 4.80 (0.90–27.10) | 6.17 (1.30–82.9) | $r = 0.72^a$ | $t = 4.0$ $P = 0.001$ |

[a]$P < 0.001$.

**Figure 1** The relationship between the *LI*, $T_S$ and $T_{pot}$ values from 139 samples measured at two different institutions by different observers. The intersecting lines represent the median values. The correlation coefficients ($r$) were 0.86 (*LI*), 0.52 ($T_S$) and 0.72 ($T_{pot}$).

**Figure 2** The relationship between the $T_{pot}$ values under 20 days measured at two different institutions by different observers.

*Correction factor*

In order to reduce the degree of variation seen, attempts were made to correct for any factor which may differ between institutions or observers. The 42 samples which had been run on all of the various observer/laboratory combinations (Table II) were compared against a standard laboratory/observer combination which was *GW* at the Gray Lab. This combination was chosen as the most established and experienced one.

Three approaches were assessed on their ability to reduce the inter-institutional and -observer variation.

(i) Addition or subtraction of a factor equal to the difference between the mean for each of the laboratory/observer combinations and that of the standard combination.
(ii) Division of each value by a factor equal to the ratio of the mean for each laboratory/observer combination and the mean for the standard.
(iii) A linear regression line was fitted for each of the laboratory/observer combinations of the value for that combination against the corresponding value as the standard. These regression lines were used to correct each of the experimental values.

**Table II** *IUdR* related parameters as measured by the various observer/laboratory combinations on the same 42 samples

| | *LI* (mean ± s.d.) | *RM* (mean ± s.d.) | $T_s$ (mean ± s.d.) | $ln(T_s)$ (mean ± s.d.) | $T_{pot}$ (mean ± s.d.) | $ln(T_{pot})$ (mean ± s.d.) |
|---|---|---|---|---|---|---|
| MW/Gray | 13.1, 8.1 | 0.77, 0.08 | 13.5, 6.1 | 2.5, 0.4 | 5.1, 4.0 | 1.41, 0.7 |
| GW/Gray | 12.6, 7.8 | 0.72, 0.07 | 16.9, 7.3 | 2.8, 0.4 | 7.0, 6.3 | 1.7, 0.7 |
| MW/Paterson | 13.8, 8.9 | 0.77, 0.09 | 13.7, 5.2 | 2.5, 0.4 | 4.9, 3.6 | 1.4, 0.6 |
| CW/Paterson | 13.6, 8.3 | 0.70, 0.09 | 20.9, 12.0 | 2.9, 0.6 | 6.6, 4.3 | 1.7, 0.5 |

**Table III** Inter-observer comparison

| *Parameter* | *Observer 1, mean (range)* | *Observer 2, mean (range)* | *Correlation* | *Paired t test* | |
|---|---|---|---|---|---|
| *Gray lab., n = 45* | | | | | |
| D.I. | 1.23 (1.00–1.94) | 1.23 (1.00–1.92) | $r = 1.00$[a] | $t = 0.6$ | NS |
| L.I. (%) | 13.1 (1.20–28.7) | 12.7 (0.7–29.8) | $r = 0.99$[a] | $t = 2.2$ | $P = 0.04$ |
| R.M. | 0.77 (0.57–0.95) | 0.71 (0.57–0.97) | $r = 0.63$[a] | $t = 6.1$ | $P < 0.001$ |
| $T_s$ (hours) | 14.1 (4.21–46.4) | 17.7 (5.2–47.2) | $r = 0.67$[a] | $t = 5.7$ | $P < 0.001$ |
| $T_{pot}$ (days) | 5.15 (1.12–21.9) | 8.6 (1.3–82.9) | $r = 0.87$[a] | $t = 2.2$ | $P = 0.03$ |
| *Paterson Inst., n = 68* | | | | | |
| D.I. | 1.18 (1.00–1.96) | 1.19 (1.00–1.80) | $r = 0.84$[a] | $t = 0.95$ | NS |
| L.I. (%) | 14.8 (0.9–34.80) | 14.5 (0.9–31.20) | $r = 0.94$[a] | $t = 1.0$ | NS |
| R.M. | 0.75 (0.56–0.98) | 0.69 (0.54–0.89) | $r = 0.67$[a] | $t = 6.7$ | $P < 0.001$ |
| $T_s$ (hours) | 16.2 (4.6–53.00) | 22.3 (5.2–80.0) | $r = 0.67$[a] | $t = 4.4$ | $P < 0.001$ |
| $T_{pot}$ (days) | 5.3 (1.1–27.10) | 6.7 (1.8–33.2) | $r = 0.73$[a] | $t = 5.0$ | $P < 0.001$ |

[a]$P < 0.001$.

**Figure 3** The relationship between the *LI*, $T_S$ and $T_{pot}$ values calculated by two observers using data obtained from 45 samples at one institution (Gray Lab.). The intersecting lines represent the median values. The correlation coefficients (*r*) were 0.99 (*LI*), 0.67 ($T_S$) and 0.87 ($T_{pot}$).

**Figure 4** The relationship between *LI*, $T_S$ and $T_{pot}$ values for 30 samples run on two separate occasions on the same machine and analysed by the same observer. The intersecting lines represent the median values. The correlation coefficients (*r*) were 0.87 (*LI*), 0.57 ($T_S$) and 0.87 ($T_{pot}$).

Correction factors (i) and (ii) both significantly reduced the laboratory and observer variances for *LI* and $T_{pot}$, but not those of *RM* and $T_S$. Correction factor (ii) reduced the inter-institutional coefficient of variation from 30 to 2% and the inter-observer variation from 112 to 62%. Correction factor (iii) did not appear to reduce the variation of any of the parameters. Even after correcting the data, the laboratory and observer differences remained significant except $T_{pot}$ where laboratory difference was not significant. *RM* and $T_S$ showed no reduction in either inter-institutional or -observer variability after such corrections.

*Reproducibility*

In order to measure the reproducibility of the results generated by the same machine on the same samples, the same 30 samples were run on both of the flow cytometers on two separate occasions following storage at 4°C for 1 week. The cytometers were re-calibrated before each run of 30 samples. The resulting data were then analysed by the same individual on both occasions. There was good correlation between the two samples (e.g.: $T_{pot}$ values, $r = 0.74$, $P < 0.001$ Gray Lab; $r = 0.87$, $P < 0.001$ Paterson Inst.) with no statistical difference present in paired analyses of any of the parameters (Table IV and Figure 4).

*Global analysis*

Table V demonstrates the coefficients of variation for each of the *IUdR* related parameters calculated using hierarchical *ANOVA*. This is a global analysis on 457 sets of data that includes:

(i) 172 samples run at the Paterson Institute and 124 samples run at the Gray Laboratory;
(ii) 62 sets of data reanalysed at the Paterson by two observers and 39 sets that were reanalysed at the Gray Lab.;
(iii) 30 samples that were re-run on two occasions on each flow cytometer.

The 'observer' category represents the variation seen between the different observers and is fitted first because it displays the largest variance in $T_{pot}$. *LI* has a larger variance in the institute category, which suggests that for this parameter, there is greater variation between machines than individual observers. All the following categories (institution, sample and re-run) are then treated in order, with the variation due to the preceding categories having been accounted for. The variation seen in the value for *LI* is 143% once the variation due to different observers and institutions have been included. Both $T_S$ and $T_{pot}$ showed skewed distributions

**Table IV** Machine reproducibility

| *Parameter* | *Run 1, mean (range)* | *Run 2, mean (range)* | *Correlation (significance)* |
|---|---|---|---|
| *Gray lab., n = 30* | | | |
| DI | 1.19 (1.00–1.77) | 1.21 (1.00–1.69) | $r = 1.00$ $P < 0.001$ |
| LI (%) | 16.1 (2.9–34.6) | 15.9 (3.2–29.8) | $r = 0.98$ $P < 0.001$ |
| RM | 0.73 (0.57–0.89) | 0.73 (0.59–0.92) | $r = 0.40$ $P = 0.012$ |
| $T_s$ (hours) | 17.6 (6.2–53.1) | 17.4 (8.8–47.2) | $r = 0.57$ $P < 0.001$ |
| $T_{pot}$ (days) | 4.6 (1.7–17.5) | 4.7 (1.3–19.2) | $r = 0.74$ $P < 0.001$ |
| *Paterson lab., n = 30* | | | |
| DI | 1.20 (1.00–1.64) | 1.19 (1.00–1.64) | $r = 0.88$ $P < 0.001$ |
| LI (%) | 18.3 (3.9–34.8) | 18.3 (4.4–34.7) | $r = 0.87$ $P < 0.001$ |
| RM | 0.75 (0.63–0.92) | 0.77 (0.63–0.91) | $r = 0.54$ $P = 0.001$ |
| $T_s$ (hours) | 15.8 (6.5–25.0) | 14.9 (6.1–29.5) | $r = 0.76$ $P < 0.001$ |
| $T_{pot}$ (days) | 3.5 (0.9–9.1) | 3.3 (0.9–9.0) | $r = 0.87$ $P < 0.001$ |

**Table V** Coefficients of variation (%) of the IUdR related parameters

| | *LI* | *RM* | $T_s$ | $ln(T_s)$ | $T_{pot}$ | $ln(T_{pot})$ |
|---|---|---|---|---|---|---|
| Observer | 91.4 | 39.2 | 165.9 | 48.4 | 181.6 | 131.1 |
| Institute | 132.0 | 20.0 | 98.3 | 44.3 | 104.4 | 44.0 |
| Sample | 142.5 | 24.3 | 101.4 | 37.1 | 241.5 | 103.2 |
| Re-runs | 27.8 | 7.8 | 39.9 | 10.3 | 41.5 | 24.9 |

and therefore a logarithmic transformation was applied to these variables. The un-transformed values are also shown to allow comparison with published results from other studies. This analysis indicates that there are highly significant differences between different samples once the variation due to different observers and institutions have been taken into account. The estimates of ploidy (diploid *vs* aneuploid) show no significant differences between observers ($P = 0.11$) or institutions ($P = 0.12$), but highly significant differences between individual tumours ($P < 0.001$). Once these effects are allowed for, 92% of the samples are correctly categorised into above or below the median value.

### Discussion

Cell proliferation kinetics have been shown to be a prognostic factor for several types of cancer as reviewed by Tubiana & Courdi (1989). These studies have been carried out using either tritiated thymidine labelling or flow cytometric measurements of *S*-phase fractions. The first method is time consuming and has limited clinical application due to the requirement for the administration of a radioactive isotope. The second method may over-estimate proliferative capacity as there is evidence that cells can arrest in phases other than $G_1$ (Drewinko *et al.*, 1984). Furthermore, it is difficult to exclude the normal stromal nuclei from the analysis. The development of a monoclonal antibody to the halogenated pyrimidines *IUdR* and *BUdR* (Gratzner, 1982), allowed the establishment of a rapid technique for the simultaneous measurement of tumour DNA content, labelling index (*LI*) and an estimation of the duration of *S*-phase ($T_S$). These measurements provide the basis for the calculation of $T_{pot}$. Although there are several clinical studies published using this technique on a variety of tumour types (Wilson *et al.*, 1988; Begg *et al.*, 1990; Rew *et al.*, 1991; Riccardi *et al.*, 1991; Rew *et al.*, 1992), the reliability and reproducibility of the method has yet to be examined in detail.

It has been demonstrated that the measurement of ploidy may be performed accurately both between and within institutions (Wheeless *et al.*, 1991). This study confirms the finding in that there are no significant differences in ploidy determinations between the present institutions, individuals analysing the same stored data and in samples re-run on the same machine. As regards the other *IUdR*-related parameters (*LI*, *RM*, $T_S$ and $T_{pot}$), these measurements were not as closely reproducible.

A comparison of the values obtained by running samples on two separate machines and analysed by different observers showed that there were significant differences in the measured parameters. However, most results were well correlated, i.e.: there was good agreement that a given sample had a high or low *LI*, *RM*, $T_S$ or $T_{pot}$ value. The best correlations were seen in the measurements of *LI* ($r = 0.86$) and $T_{pot}$ ($r = 0.72$). However, correlations for *RM* (0.48) and $T_S$ (0.52) were not as strong.

Significant but less marked variation was seen between observers analysing the same stored data. In this case values for *LI* were not significantly different and the correlation coefficients obtained were higher than for the inter-institutional comparison. The differences seen in measured values for *RM*, $T_S$ and $T_{pot}$ are likely to represent discrepancies in delineating $G_1$, *S* and $G_2$. All three of the data analysers used the same previously agreed criteria on where to define the various regions, and all obtained $G_2$ from the *2D* histograms. It may be that the degrees of correlation obtained could be improved, either by arbitrarily defining $G_2$ as simply twice $G_1$ or by using the *1D* histogram, which often displays $G_2$ more visibly. Defining the $G_2$:$G_1$ ratio as two has been used in previous studies (e.g. Begg *et al.*, 1988). In the present study, this had the effect of lengthening the calculated $T_{pot}$ values and made the determination of $T_{pot}$ impossible in two cases as the *RM* value dropped below 0.5. The scatter, as indicated by the correlation between two individuals, was greater if the $G_2$:$G_1$ ratio was assumed to be 2 (e.g. $r = 0.70$ *vs* 0.92). This indicates that arbitrarily attributing a value to $G_2$ makes the measurement less accurate. It should be noted that there have been attempts to improve the calculation of $T_S$ by a more rigorous mathematical method (White & Meistrich, 1986; White, 1989; White *et al.*, 1990). However, comparisons of these sophisticated approaches with the more simple *RM* method (Begg *et al.*, 1985) suggest that the difference in $T_{pot}$ calculated is small and identification of the appropriate regions is easier with the *RM* method (Wilson & McNally, 1991).

Although the actual values obtained were significantly different between different institutions and observers, there was good agreement in the ranking of the results. Attempts were made to reduce the degree of variation seen in the comparisons using a correction factor. This was considered and three mathematical models were assessed using pooled results of 42 samples which had been run on all of the various observer/laboratory combinations. The greatest reduction in variance was seen after correction by a factor corresponding to the ratio between any given observer/institute combination and a 'gold standard' which was *GW* at the Gray Laboratory. This was chosen due to the greatest experience of that combination. By doing this it was possible to reduce the $T_{pot}$ *CV* between institutes from 30 to 2%, whereupon the variation was not significantly different. Reduction in the inter-observer variation was from 112 to 62% but this remained a highly significant degree of variability ($P < 0.001$). These results indicate that, whilst there is good agreement between different institutions and observers analysing stored data in deciding the ranking of $T_{pot}$ values, the actual numerical values for $T_{pot}$ are significantly different. The former may be totally correctable, the latter only partially i.e. it is not possible to use a mathematical factor to remove all of the variation between laboratories and observers. This demonstrates the difficulties of pooling data and supports the concept that in a comparative cell kinetic study a single individual should be responsible for the preparation and analysis of the samples using a single flow cytometer.

The degree of machine reproducibility was tested by re-analysing nuclei from 30 specimens stored for 1 week at 4°C in the dark. There were no significant differences in the measured values obtained, illustrating both the reproducibility of the technique and the efficacy of sample storage for a short period of time. A further source of variability is the presence of heterogeneity with in individual tumours. This particular problem has been previously addressed (Begg *et al.*, 1988; Rew *et al.*, 1991) and forms the basis of a paper presently being produced.

The median $T_{pot}$ value has been previously used as a watershed to determine if a tumour is a fast or slow proliferator (Begg *et al.*, 1990). Despite the variation demonstrated in this study, the percentage of samples where there was agreement in their position above or below the median was 77% between different observers, 89% between different institutions and 87% between different runs on the same machine/observer combination. Eighty per cent of the samples were consistently placed either above or below the median, irrespective of which particular observer/laboratory combination was used.

In conclusion, we have demonstrated that the *IUdR* technique for measuring tumour proliferation is reproducible. The variation in the assay is far less than that seen between individual tumours. Correlations were seen between the measurements of obtained by different individuals and on separate machines. These were good for *DI*, *LI* and $T_{pot}$ and weakest for *RM* and $T_S$ suggesting that the main source of variability is from the *RM* measurements which are used to calculate $T_S$ and subsequently $T_{pot}$. Direct comparisons of the actual measured values showed that there were highly significant differences in the values obtained between institutes and observers. Thus, whilst the institutions/observers agreed in terms of classifying values as high or low, the ranges of values were different. These data suggest that large clinical trials using $T_{pot}$ should use a single centre and a single experienced individual to prepare, run and analyse samples.

The authors gratefully acknowledge the technical support received from the flow cytometric facilities of the Gray Laboratory and the Paterson Institute and discussion with Drs J. Hendy and E. Anderson. The work was funded by the Christie Hospital Endowment Fund and the Cancer Research Campaign.

### References

BEGG, A.C., HOFLAND, I., MOONEN, L., BARTELINK, H., SCHRAUB, S., BONTEMPS, P., LE FUR, R., VAN DEN BOGAERT, W., CASPERS, R., VAN GLABBEKE, M. & HORIOT, J.C. (1990). The predictive value of cell kinetic measurements in a European trial of accelerated fractionation in advanced head and neck tumours: an interim report. *Int. J. Rad. Oncol. Biol. Phys.*, **19**, 1449–1453.

BEGG, A.C., MCNALLY, N.J., SHRIEVE, D.C. & KARCHER, H. (1985). A method to measure the duration of DNA synthesis and the Potential Doubling Time from a single sample. *Cytometry*, **6**, 620–626.

BEGG, A.C., MOONEN, L., HOFLAND, I., DESSING, M. & BARTELINK, H. (1988). Human tumour cells kinetics using a monoclonal antibody against iodoeoxyuridine: intra-tumoural sampling variations. *Radiother. Oncol.*, **11**, 337–347.

DISCHE, S. & SAUNDERS, M.I. (1989). Continuous, hyperfractionated accelerated radiotherapy (CHART). *Br. J. Cancer*, **59**, 325–326.

DREWINKO, B., YING YANG, L., BARLOGIE, B. & TRUJILLO, J.M. (1984). Cultured human tumour cells may be arrested in all stages of the cell cycle during stationary phase: demonstration of quiescent cells in G1, S and G2 phase. *Cell Tiss. Kinet.*, **17**, 453–463.

GRATZNER, H.G. (1982). A monoclonal antibody to 5-bromodeoxyuridine and 5-iododeoxyuridine: a new reagent for detection of DNA replication. *Science*, **218**, 474–475.

REW, D., WILSON, G., TAYLOR, I. & WEAVER, P. (1991). Measurement of *in vivo* proliferation in human colorectal tumours. *Br. J. Surg.*, **78**, 60–66.

REW, D.A., CAMPBELL, I.D., TAYLOR, I. & WILSON, G.D. (1992). Proliferation indices of invasive breast carcinomas after *in vivo* 5-bromo-2′-deoxyuridine labelling: a flow cytometric study of 75 tumours. *Br. J. Surg.*, **79**, 335–339.

RICCARDI, A., GIORDANO, M., DANOVA, M., GIRINO, M., BRUGNATELLI, S., UCCI, G. & MAZZINI, G. (1991). Cell kinetics with *in vivo* bromodeoxyuridine and flow cytometry: clinical significance in acute non-lymphoblastic leukaemia. *Eur. J. Cancer*, **27**, 882–887.

STEEL, G.G. (1977). *Growth Kinetics of Tumours*. Oxford University Press: London.

TUBIANA, M. & COURDI, A. (1989). Cell proliferation kinetics in human solid tumours: relation to probability of metastatic dissemination and long-term survival. *Radioth. & Oncol.*, **15**, 1–18.

WHEELESS, L.L., COON, J.S., COX, C., DEITCH, A.D., DEVERE WHITE, R.W., FRADET, Y., KOSS, L.G., MELAMED, M.R., O'CONNELL, M.J., REEDER, J.E., WEINSTEIN, R.S. & WERSTO, R.P. (1991). Precision of DNA flow cytometry in inter-institutional analyses. *Cytometry*, **12**, 405–412.

WHITE, R.A. (1989). Computing multiple cell kinetic properties from a single time point. *J. Theor. Biol.*, **141**, 429–446.

WHITE, R.A. & MEISTRICH, M.L. (1986). A comment on 'A method to measure the duration of DNA synthesis and the potential doubling time from a single sample'. *Cytometry*, **7**, 486–490.

WHITE, R.A., TERRY, N.H.A., MEISTRICH, M.L. & CALKINS, D.P. (1990). Improved methods of computing potential doubling time from flow cytometric data. *Cytometry*, **11**, 314–317.

WILSON, G.D. & MCNALLY, N.J. (1991). Measurement of cell proliferation using bromodeoxyuridine. In: *Cell Proliferation in Clinical Diagnosis*, pp. 113–139. Hall, P.A., Levison, D.A. & Wright, N.A. (eds). Springer-Verlag, London.

WILSON, G.D., MCNALLY, N.J., DUNPHY, E., KARCHER, H. & PFRAGNER, R. (1985). The labelling index of human and mouse tumours assessed by bromodeoxyuridine staining *in vitro* and *in vivo* and flow cytometry. *Cytometry*, **6**, 641–647.

WILSON, G.D., MCNALLY, N.J., DISCHE, S., SAUNDERS, M.I., DES ROCHERS, C., LEWIS, A.A. & BENNETT, M.H. (1988). Measurements of cell kinetics in human tumours *in vivo* using BUdR incorporation and flow cytometry. *Br. J. Cancer*, **58**, 423–431.